# Left Amygdala and Putamen Activation Modulate Emotion Driven Decisions in the Iterated Prisoner’s Dilemma Game

**DOI:** 10.3389/fnins.2019.00741

**Published:** 2019-07-17

**Authors:** Iveta Eimontaite, Igor Schindler, Matteo De Marco, Davide Duzzi, Annalena Venneri, Vinod Goel

**Affiliations:** ^1^Department of Psychology, University of Hull, Hull, United Kingdom; ^2^Department of Neuroscience, The University of Sheffield, Sheffield, United Kingdom; ^3^IRCCS San Camillo Hospital Foundation, Venice, Italy; ^4^Department of Psychology, York University, Toronto, ON, Canada; ^5^Capital Normal University, Beijing, China

**Keywords:** prisoner’s dilemma, sympathy, anger, amygdala, putamen, cooperation, decision-making

## Abstract

Although economic decision-making is commonly characterized as a purely rational phenomenon, it is clear that real-world decision-making is influenced by emotions. Yet, relatively little is known about the neural correlates of this process. To explore this issue, 20 participants underwent fMRI scanning while engaged in the Prisoner’s Dilemma game under partner-directed sympathy, anger and neutral emotion conditions. Participants were most and least likely to cooperate after sympathy and anger induction, respectively, with the neutral condition eliciting intermediate cooperation rates. Moreover, the sympathy condition elicited quicker responses for cooperation than defection choices, whereas this pattern was reversed in the anger and neutral conditions. Left amygdala activation showed a positive correlation with cooperation rates and self-reports of partner directed sympathy in the sympathy condition. In the anger condition, left putamen activation was positively correlated with cooperation rates and negatively correlated with self-reports of partner directed anger strength. These findings indicate that while the left amygdala activation may be indicative of emotion enhancement and increase of cooperative behavior, the left putamen may help to suppress an emotion to overcome anger and engage in cooperation.

## Introduction

Human choice often involves tension between cooperation and non-cooperation. Actions to combat climate change provide a relevant real-world example. As a society we and our children would be much better off if we all cooperated in reducing carbon emissions (and jointly bear the costs). However, as an individual, if I bear the cost and reduce my carbon footprint (cooperate), and my neighbor continues to pollute (defect), he will reap a greater benefit than myself. If, however, I choose to continue polluting (defect), and my neighbor bears the cost of reducing carbon emissions (cooperate), I will reap the greatest benefit. If we both choose to continue polluting (defecting), we will both suffer equally. These types of choices are often formulated and studied in the laboratory as variations of the Prisoner’s Dilemma game (Oskamp and Perlman, 1965).

For much of the 20th century, the dominant view of humans, embodied in the “homo economicus” model was as a utility maximiser as a consumer, and a profit maximiser as a producer. On this model, decision-makers will exhibit perfect self-interested rationality and select the choice most advantageous for them. What makes such game theoretic tasks interesting is that the advantageous choice is dependent upon predicting the choice made by your opponent. Economic theory argues that defecting or not cooperating with your partner in the iterated Prisoner’s Dilemma game can be consistent with utility maximizing behavior (Neyman, 1985). Data on such tasks show that participants will typically cooperate 40% of the time, while defecting approximately 60% of the time (Jones et al., 1968; Bó and Fréchette, 2011).

The “homo economicus” model is slowly changing as we begin to accept and accommodate the reality that various factors including emotions (Goel and Dolan, 2003; Goel and Vartanian, 2011; Halperin et al., 2013; Smith et al., 2014, 2015; Goel et al., 2017; Levine et al., 2017; Eimontaite et al., 2018), reward processing (Sanfey et al., 2003), Theory of Mind (Camerer, 2003) and individual differences in cognitive inhibition (De Neys et al., 2011), social orientation (Emonds et al., 2014), and trust (Chaudhuri et al., 2002; Lambert et al., 2017) modulate our decision-making. In fact, trust and cooperation are hard to separate and quite often these terms are used interchangeably while investigating social interaction games (Yamagishi et al., 2005). However, the attempts to separate cooperation and trust show that cooperation leads to trust (Chaudhuri et al., 2002; Yamagishi et al., 2005). Our focus here is on the effect of emotions on rational choice in the Prisoner’s Dilemma game and development of cooperation as predecessor of trust in social interactions.

Common sense tells us that emotions should drive decisions by modulating subjective experiences (Scherer, 1982, 2005). Behavioral data, unsurprisingly, indicate that sympathy can encourage higher cooperation levels, even if it is costly/detrimental to the decision-maker (Bloom, 2017). Anger can trigger higher defection rates, again, even at a cost to the decision-maker (Bosman and Van Winden, 2002; Ben-Shakhar et al., 2004; Duersch and Servátka, 2007). In a study by Kopelman et al. (2006), participants in the role of sellers made higher demands while interacting with buyers displaying negative emotions by asking higher prices, and provided shorter warranty periods, etc. Buyers were less likely to sign a deal in the negative emotion condition compared to positive and neutral emotion conditions (Kopelman et al., 2006). The same pattern of behavior is observed in the iterated Prisoner’s Dilemma with anger and sympathy emotions felt toward the other: sympathy toward the opponent increases cooperation, while anger toward the opponent increases defection compared to the neutral condition (Eimontaite et al., 2013). On the other hand, it is not only the valence of the emotion which needs to be considered, but also the motivation which is triggered by induced emotion. Engelmann and Hare (2018) review studies where withdrawal-related emotional states, such as sadness, fear and empathy, lead toward risk averse choices, while approach-related emotions, such as anger, lead to more risky decisions. These results, as Engelmann and Hare (2018) note, are also reflected in the neuroimaging study findings: choices under safety show activation in the ventromedial prefrontal cortex and ventral striatum, but not the insula. Yet, insula activation is evident under conditions of sadness and the perception of fairness.

Although emotions are important in decision-making, they are not the only factors determining the choice one will make. The perception of the possible rewards/gains or losses also affect decision-making processes (McCabe et al., 2001; Sanfey et al., 2003; King-Casas, 2005). Reward processing in the brain is marked by striatum activation (including putamen and caudate) and in economic games has shown an increase in activation associated with winnings (Elliott et al., 2003; Haruno, 2005; Hsu et al., 2008), and the decrease in activation with losses (Verney et al., 2003; Bjork, 2004).

Strategic thinking is also an important factor in decision-making and seems to be represented by medial prefrontal cortex activation (Blair et al., 1999; Frith, 2001; McCabe et al., 2001; Decety et al., 2004). High co-operators in the Trust Game showed stronger medial prefrontal cortex activation whilst interacting with human opponents as opposed to interacting with a computer. Yet for high defectors, activation of this region did not depend on the type of the opponent – human or computer (McCabe et al., 2001). Furthermore, deciding to trust individuals from the same racial group or not involved the striatum and amygdala (Stanley et al., 2012). In particular, striatum activation was recorded during representation of race-based reputations that shape trust decisions, while the amygdala was involved in processing emotionally relevant social group information. The amygdala is also critical for forming trust: patients with lesions to the amygdala tended to increase trust in response to betrayals in the Trust Game, while neurologically normal adults and patient controls show a decrease in trust after betrayals (Koscik and Tranel, 2011).

Tasks like the Prisoner’s Dilemma can be presented either as single shot trials or multiple trials involving extended social interaction. The latter, iterated version of the task, presents the outcome of the interaction after each trial. This introduces complexity in terms of social context and reputation building, requiring additional strategizing (Camerer, 2003; Cuesta et al., 2015; Levine et al., 2017; Li et al., 2017). Reputation building involves monitoring the choices of your opponent in the context of your choices. If you cooperate on a particular trial, but your opponent chooses to defect, this will affect your decision on subsequent trials. But it will also have an emotional impact in terms of making you angry, upset, disappointed, or feeling cheated. In such a case, it is not clear how one would separate the effects of reputation building from emotions. Separating the influences of the emotional state of the decision-maker from strategic thinking in decision-making processes would allow further understanding of how various social influences shape decision-making. Separating the influences of emotional states of the decision-maker from strategic thinking in decision-making processes would allow further understanding of how various social influences shape decision-making. Some research has used iterated single shot games with unknown opponents to avoid reputation building effects (Ramsøy et al., 2015; Macoveanu et al., 2016), and this paradigm allows to investigate decision-making without prior emotion induction. However, adding emotion induction unrelated to the interaction in the game would be complicated in the context of iterated single shot games and difficult for the participant to keep track of.

The goal of the present study was to identify brain regions associated with decision-making in the Prisoner’s Dilemma under the influence of three partner-directed emotion conditions: sympathy, anger, and neutral. Several previous neuroimaging studies have explored the effect of emotions on decision-making in the Prisoner’s Dilemma game. In a study by Singer et al. (2004) participants and their opponents (who were not real) had to interact on a Prisoner’s Dilemma type game. They had two conditions – make decisions by themselves (intentional) or follow predetermined decision by a computer. After the interaction, participants were asked to evaluate the other players and the results revealed sympathetic responses with cooperative opponents, and anger toward defecting opponents when these decisions were intentional (participants decided by themselves and were not determined by computer). In a follow-up study, after interaction in the Prisoner’s Dilemma game, participants had to observe pain induction to cooperative opponents and this increased their anterior insula and anterior cingulate cortex activation (Singer et al., 2006). However, during the same pain induction to the unfair opponent, male participants showed increased activity in nucleus accumbens.

Rilling et al. (2008) looked at the interaction between reciprocated and unreciprocated cooperation in the iterated Prisoner’s Dilemma game. In particular, opponent’s defection after participants cooperation showed greater activation in bilateral anterior insula, left hippocampus and left amygdala, while bilateral ventral striatum showed deactivation. Furthermore, unreciprocated cooperation after previous cooperation compared to defection showed increased activity in anterior insula and left hippocampus. These results indicate that these areas are responsive to unreciprocated cooperation and anger emotion as reported by participants in a post-experiment questionnaire. Although these studies provide some insight into how emotions affect decision-making in socio-economic games, the emotion is triggered by the game play and it is hard to disengage whether emotions were driving the decision or they were incidental to the outcomes.

Our study differs from previous efforts in three respects. First, we avoided the potential confound of reputation building by keeping participants blind to the outcome of each trial in addition to avoiding one shot games. Second, participants’ knowledge of the other player was built by emotion induction prior to the interaction in the Prisoner’s Dilemma game. That is, emotions were triggered by an event that was incidental to the decision situation, but the emotion was decision-relevant as it was triggered by and directed toward their opponent in the Prisoner’s Dilemma game. Finally, we compared the effect of two distinct emotions, sympathy and anger, in a within-subject design, allowing us to investigate cooperation and defection choices while controlling for individual differences. We predicted participants would show more cooperation in the sympathy condition compared to the neutral condition, and more defection in the anger compared to the neutral condition (Eimontaite et al., 2013). At the neural level we were interested in the interaction between emotion and choice and expected activation in the brain areas previously identified to be involved in emotional stimuli processing, processing of trustworthiness and decision-making. In particular, we predicted amygdala activation during processes where participants would embrace emotions and emotionally relevant information about individuals (Koscik and Tranel, 2011; Stanley et al., 2012), and striatum, and in particular putamen, activation for overcoming emotion effects (Padmala and Pessoa, 2010; Cutler and Campbell-Meiklejohn, 2019). That is, increased cooperation in sympathy condition would result in activation in the amygdala, while decreased defection in anger condition would show putamen involvement.

## Materials and Methods

### Participants

Twenty-two Italian health care professionals employed at the IRCCS San Camillo Hospital Foundation (Venice, Italy) voluntarily took part in the study. Two participants were removed due to awareness of the deception and extensive head movement in the scanner, leaving 20 participants (6 males, 14 females) in the final analysis. Mean age of the participants was 29 years (*SD* = 5.68), and mean education was 16.4 years (*SD* = 3.54). Participants had normal or corrected to normal vision, 18 were right-handed. The study was approved by the University of Hull (United Kingdom) and the IRCCS San Camillo (Italy) ethics committees. Each participant provided written informed consent.

### Task

**FIGURE 1:**
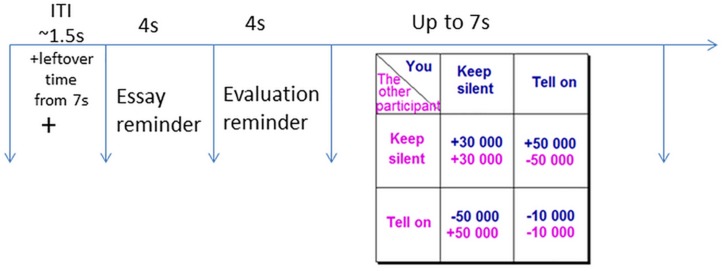
A single trial of the Prisoner’s Dilemma would start with a fixation cross (1.5 s), followed by a reminder of the other “participant’s” essay and evaluation (each for 4 s). In the end the participant would make a choice (up to 7 s) on the Prisoner’s Dilemma outcome table by pressing either the Index finger (cooperation) or the Middle finger (defection).

The Prisoner’s Dilemma game simulated a hypothetical situation whereby you and a partner are bankers suspected in corporate malfeasance. The police interrogate both of you separately, and offer each of you the following deal: If you provide the missing facts to the police (i.e., defect on your partner), and your partner stays silent, you will get a reward of €50,000 and your partner will pay a fine of €50,000. If both of you confess (defect on each other) and fill in the facts for the police, you will both pay a fine of €10,000. If you choose to stay silent (cooperate with your partner), and your partner fills in the facts for the police (defects), he/she will receive a reward of €50,000 and you will pay a fine of €50,000. If both of you choose to stay silent (cooperate), the police will not have enough evidence to convict either of you and will be forced to pay you €30,000 each for wrongful arrest. The payoff matrix is presented in Figure 1. The payoffs are a function of, not only the participant’s selection, but also the selection of their partner. The task requires participants to make decisions that will maximize their hypothetical gains and minimize hypothetical losses. Each participant plays the game with three different partners, under three different emotion conditions.

### Emotion Inducement

Steps were also taken to make participants feel anger or sympathy toward two of the three partners and remain neutral toward the third partner prior to the commencement of the game via an essay writing and evaluation task. Participants were asked to write a short essay describing something important to them. The experimenter would take the essay out of the room, explaining that it would be given to their “partner” for comments/evaluation and that they would be required to evaluate the partner’s essays. Approximately 5 min later the experimenter would return with one of the “partner’s” essays for evaluation. After the evaluation was completed, the experimenter would take the evaluation and leave the room to retrieve the participant’s essay evaluated by their “partner” (Eimontaite et al., 2013).

In actuality, the participant was being deceived. There were no other participants. The experimenter would return with the participant’s essay, purportedly evaluated by their “partner.” These evaluations consisted of the ratings of the essays on six 9-point bipolar scales (unintelligent–intelligent; thought provoking–boring; friendly–unfriendly; illogical–logical; respectable–unrespectable; irrational–rational), along with a space for free comments. In the sympathy condition the emotion was induced with an essay written by a young person coping with cancer [modified from Harmon-Jones et al. (2003)]. In this condition the evaluation of the participant’s essay was rated neutrally (between 4 and 7 on the evaluation scales) and a hand-written positive comment “I can understand why a person would think like this” was left underneath the evaluation. In the anger condition, emotion was mainly triggered by the negative evaluation consisting of ratings that were weighted toward negative words (e.g., illogical or unacceptable). An insulting comment was also hand-written underneath the evaluation (“This is the stupidest thing I have ever read”). The essay in this condition was neutral in content, but a poorly written (grammatical mistakes, badly structured arguments). Finally, neutral emotion induction consisted of a neutral content essay, written in an unemotional and grammatically correct way, followed by a neutral (evaluations between 4 and 6) evaluation of the participant’s own essay with no hand-written comments.

The procedure was repeated three times (once per each emotion condition) and photographs of both the opponent’s essays and the evaluations of the participant’s own essay were taken to strengthen the deception (all photographs were prefabricated before the experiment). These photographs of essay and evaluation were later presented before each Prisoner’s Dilemma trial so that participants would know with whom they were interacting. Following emotion induction, participants previewed the uploaded photographs of their “partner’s” essays and the evaluations they received on their own essays, to familiarize them with the digital versions.

The essays and evaluations were hand-written on different color paper (light blue, light purple, and light green) so that participants would learn to associate a color with a particular “partner.” Colors associated with particular conditions hence the conditions were counterbalanced across participants.

### The Task Presentation

An iterated 108-trial version (36 per emotion condition) of the game was used in the experiment. Each individual trial of the game would start with a fixation cross remaining on screen for 1.5 s on average followed by the scanned essay and the evaluation from the emotion induction 4 s each, both color-coded to provide content cues; serving as a rumination helping to prolong the emotion duration (Sbarra and Emery, 2005; Verduyn et al., 2009). Finally, the payoff matrix was presented for 7 s during which participants had to choose between cooperation and defection. If the participant made their decision in less than 7 s, the remaining time was added to the inter trial interval (ITI).

The order of the emotion conditions was pseudo-randomized, allowing a maximum of three consequent trials of the same emotion condition. Six different payoff matrices were presented and the amount possible to gain and lose in each had the same proportions (3: 5: −5: −1; i.e., participant cooperates/other cooperates: +€30,000/+€30,000; participant cooperates/other defects: +€50,000/−€50,000; participant defects/other cooperates: −€50,000/+€50,000; participant defects/other defects: −€10,000/−€10,000; Figure 1). Three pre-determined outcomes of the interaction (“You get €315,500 out of overall €730,000 possible earnings,” “You get €396,000 out of overall €849,000 possible earnings,” or “You get €745,000 out of overall €900,000 possible earnings”) were counterbalanced between three runs. These outcomes were presented only after 36 trials to avoid a reputation effect, and were independent of the participants’ responses (Figure 1). Participants were not provided information about the opponent earnings. The dependent measure was the mean number of defection and cooperation per emotion condition.

In addition to the iterated Prisoner’s Dilemma game participants completed a self-report emotion questionnaire to evaluate the success of emotion induction [adapted from Harmon-Jones et al. (2003), and Harmon-Jones and Sigelman (2001)]. Words being semantically related to sympathy, compassion, and sadness were pooled into a sympathy word group (Cronbach’s Alpha 0.914, *n* = 17). Similarly, words indicative of anger and fear emotions were combined to an anger emotion word list (Cronbach’s Alpha 0.875, *n* = 17), and the neutral emotion word list contained adjectives associated with positive affect (Cronbach’s Alpha 0.834, *n* = 18). Further, a mixed ANOVA confirmed that each emotion was successfully induced as planned: in the sympathy condition, the sympathy word group was rated highest as well as the anger word group in the anger emotion condition (Supplementary Materials).

### Procedure

Before signing informed consent and agreeing to take part in the study, participants were informed that the purpose of the study is to investigate various reasoning processes. They were told that they will need to interact with other individuals in this study on some of the tasks, however, other tasks will be completed just on their own. After this, participants took part in the essay writing/emotion induction task. After emotion induction, participants were taken to the fMRI room, where they were reminded of the rules of the Prisoner’s Dilemma before playing it. The experiment consisted of three runs with 36 trials per run (12 trials of sympathy, 12 of anger, and 12 of neutral emotion condition). Each run lasted for 11.5 min. Participants did not receive the reimbursement depending on their performance in the Prisoner’s Dilemma game. After the scanning procedure, participants filled in the Self-Report Emotion Questionnaire. Finally, questions establishing the participant’s belief in the deception were asked and the full debrief was given providing the true aims of the experiment.

### fMRI Acquisition

Scanning was performed at the IRCCS San Camillo using a 1.5T Phillips Achieva MRI scanner operated with a Sense eight channel head coil. The experiment was divided into three functional runs, with time to rest between runs. Functional scans were acquired by using manufacturers standard single shot EPI sequence [TR = 2060 ms, echo time (TE) = 45 ms, flip angle = 90°, 25 slices, slice thickness = 5 mm, no gap, matrix size 80 × 80, voxel size 2.88 × 2.88 × 5 mm, FOV = 230 × 230 mm]. At the start of the scanning each participants’ fieldmap was acquired (T1 weighted fast field echo sequence, TE long = 7.6 ms, TE short 4.9 ms, slice thickness 5 mm, matrix size 72 × 60, no gap, voxel size 0.8 × 0.8 × 5 mm). Fieldmaps were used to correct EPI images for static geometric distortions caused by susceptibility-induced field inhomogeneities and head movement (Andersson et al., 2001; Hutton et al., 2002). To aid intersubject registration, at the end of each scanning session, a 3D T1-weighted structural scan was acquired for each participant (Fast field gradient echo sequence, TR = 7.4 ms, TE = 3.4 ms, 280 slices, slice thickness = 0.6 mm, matrix 240 × 240, voxel size 1.04 × 1.06).

### fMRI Analysis

Image pre-processing and data analysis were carried out using Statistical Parametric Mapping software in Matlab 2016a (SPM12; Wellcome Centre for Human Neuroimaging at UCL). The first 6 dummy volumes of each run were discarded to allow for T1 equilibration, and then the EPI images were corrected for geometric distortions caused by susceptibility-induced field inhomogeneities. Field maps were first brain extracted using FSL BET (Smith, 2002) and then processed for each participant using the FieldMap toolbox in SPM (Hutton et al., 2004). The EPI images were then realigned and unwarped (Andersson et al., 2001). Each participant’s structural image was coregistered to the mean of the motion-corrected functional images using a 12-parameter affine transformation, and segmented according to the default procedure in SPM12 (Ashburner and Friston, 2005). The spatial normalization parameters resulting from the previous step were applied to the functional images to allow for intersubject analysis. Finally, these images were smoothed using a 6 mm full width at half maximum Gaussian kernel.

For each participant, an event-related general linear model (GLM) was designed. The GLM consisted of regressors of interest: the onsets of the Prisoner’s Dilemma payoff matrix separately for cooperation and defection in each emotion condition (sympathy, anger and neutral; at the time when the payoff matrix appeared on the screen until participants made their choice and pressed the button, on average lasting 1.96 s, *SD* = 1.22). Motion parameters defined by the realignment procedure were entered as regressors of no interest, separately for each run.

Time derivatives were used and runs where either of the emotion conditions did not have a single defection or cooperation were removed (eight runs overall). Statistical parametric maps were generated from contrasts of interest: [sympathy (defection vs. cooperation) vs. neutral (defection vs. cooperation)], and [anger (defection vs. cooperation) vs. neutral (defection vs. cooperation)].

A random-effects group-level analysis using one-sample *t*-tests on the contrast images obtained from each contrast of interest for each participant was used with peak uncorrected *p* ≤ 0.005 and extent threshold of *k* = 20 (multiple testing was accounted for on cluster level based corrected *pFWE* of 0.05). This threshold was suggested to be comparable to FWE corrected thresholds according to Lieberman and Cunningham (2009), and Lieberman et al. (2009), however, further discussion by Eklund et al. (2016) shows that clusterwise inferences increase false positive error.

## Results

### Behavioral Impact of Emotion on Social Decision-Making

**FIGURE 2:**
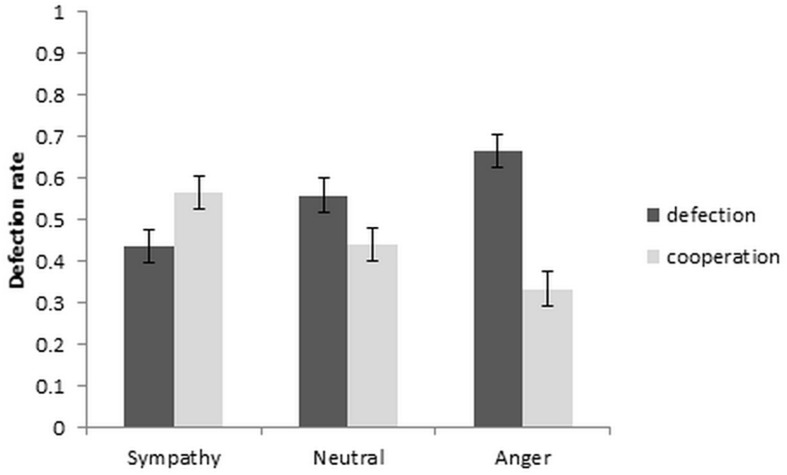
Defection and cooperation rate as a function of emotion condition (±1SEM).

To investigate the effect of sympathy, anger and neutral emotion on defection and cooperation rates, a repeated measures *ANOVA*, with independent variable of emotion condition and dependent variable of defection rate was used. The results are depicted in Figure 2. A significant repeated measures *ANOVA* [*F*(2, 36) = 6.97, *p* = 0.003, η_p_^2^ = 0.279] with *post hoc* comparisons between the emotion conditions showed that the cooperation rate increased significantly from neutral to sympathy [*t*(19) = 2.79, *p* = 0.012, *d*_z_ = 0.624], and decreased from neutral to anger at a trend level [*t*(19) = −2.07, *p* = 0.052, *d*_z_ = 0.463]. Cooperation also increased from anger to sympathy conditions [*t*(19) = 4.13, *p* = 0.001, *d*_z_ = 0.923]. Within-subject contrast showed a significant linear trend [*F*(1, 19) = 7.02, *p* = 0.016, η_p_^2^ = 0.270].

Further analysis of the reaction times with a repeated measures *ANOVA* showed the main effect of emotion as well as the emotion by choice interaction to be significant [*F*(2,38) = 6.23, *p* = 0.005, η_p_^2^ = 0.247 and *F*(2,38) = 4.55, *p* = 0.017, η_p_^2^ = 0.193, respectively]. The paired *t*-tests between the defection and cooperation choice in each emotion condition revealed significantly quicker RT’s in the sympathy condition for cooperation than defection, and also significantly quicker RT’s in defection than cooperation in the neutral condition [*t*(19) = −2.15, *p* = 0.045, *d*_z_ = 0.481 and *t*(19) = −3.20, *p* = 0.005, *d*_z_ = 0.716, respectively]. Although the reaction time in the anger condition increased from defection to cooperation choice, the increase was not significant [*t*(19) = −1.13, *p* = 0.274, *d*_z_ = 0.253; Table 1].

**TABLE 1:** Mean response time (seconds) in cooperation and defection choices (SD) and mean defection rates (SD) as a function of the emotion condition.

	**Emotion condition**		
	**Sympathy**	**Neutral**	**Anger**
Defection response time	1.90 (0.17)	1.71 (0.15)	1.57 (0.13)
Cooperation response time	1.60 (0.13)	1.93 (0.18)	1.71 (0.16)
Defection rate	0.44 (0.20)	0.56 (0.20)	0.67 (0.19)

### Imaging Results

The behavioral results indicated that anger directed at the other player increases defection, while sympathy directed at the other player increases cooperation. To isolate the neural basis of increased defection responses in the anger condition we undertook Emotion by Choice interaction analysis, comparing the BOLD signal change in the various emotion conditions as a function of defection and cooperation. We present the following three interaction contrasts (and their reverse) below: (1) [anger (defection – cooperation) – neutral (defection – cooperation)]; (2) [sympathy (defection – cooperation) – neutral (defection – cooperation)]; (3) [sympathy (defection – cooperation) – anger (defection – cooperation)]. The neural activations associated with the decision making, independent of emotions, are included in the Supplementary Materials.

#### Activation Associated With Defection in Anger Condition

**FIGURE 3:**
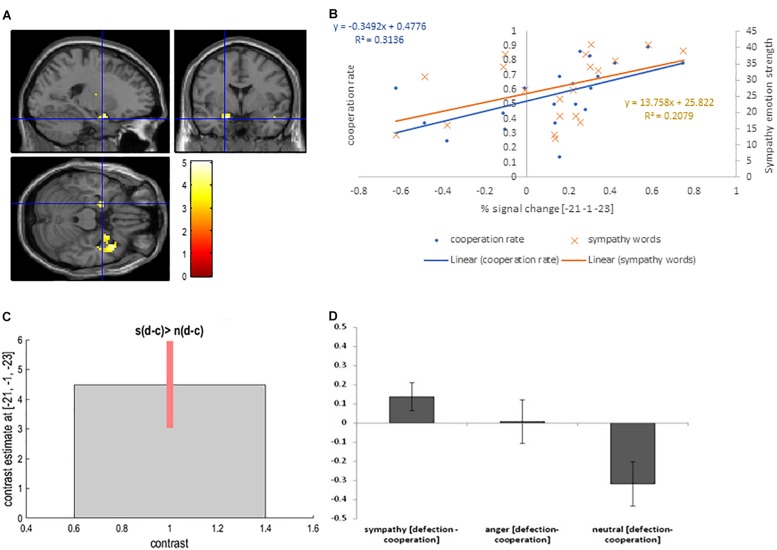
**(A)** Bilateral putamen activation overlaid on the MNI single subject template based on the interaction contrast [anger (defection – cooperation) – neutral (defection-cooperation)]; **(B)** Correlation between the interaction contrast percent signal change in the left putamen and the cooperation rate in the anger condition (blue) and correlation between the percent signal change in the left putamen and the strength of anger word rating in the anger condition (orange); **(C)** Contrast estimate and 90% Confidence Interval for interaction contrast; **(D)** Difference in percent signal change between defection and cooperation trials in sympathy, anger and neutral emotion condition for interaction contrast.

We used the contrast [anger (defection-cooperation) – neutral (defection-cooperation)] to compare the differential effects of Defection and Cooperation in Anger and Neutral conditions. It showed activation in the bilateral putamen, and the right posterior cingulate BA 23 (*P*_FWE_ < 0.05, Table 2 and Figures 3A,C,D).

**TABLE 2:** Regions of increased activation in the contrasts comparing the sympathy, anger and neutral emotion conditions between each other.

**Brain region**	**Brodmann area**	**Hemisphare**	**# of voxels**	**peak T**	**MNI coordinates**		
					**x (mm)**	**y (mm)**	**z (mm)**
**Activation associated with defection in anger condition: a(d-c)-n(d-c)**							
Sub-lobar							
Lentiform nucleus, Putamen^*^		L	56	3.45	−18	5	−8
Lentiform Nucleus, Putamen^*^		R	41	3.36	24	11	−5
Limbic Lobe							
Posterior Cingulate^∗∗^	BA 23	R	58	4.3	3	−37	22
**Activation associated with defection in Sympathy condition: s(d-c)-n(d-c)**							
Limbic Lobe							
Uncus, Superior Temporal Pole^*^	BA 28	R	321	4.84	27	5	−23
Uncus, Amygdala^∗∗^		L	108	4.66	−21	−1	−23
Cingulate Gyrus^*^	BA 23	R	691	7.33	3	−28	34
**Activation associated with defection in anger vs. sympathy condition: a(d-c)-s(d-c)**							
Sub-lobar							
Lenntiform Nucleus, Putamen^∗∗^		L	27	2.94	−18	5	−8
**Activation associated with defection in sympathy vs. anger condition: s(d-c)-a(d-c)**							
Frontal Lobe							
Medial Frontal Gyrus^∗∗^	BA 10	L	15	−4.05	−9	56	−8

^*^Cluster – level *P*_*FWE*_ ≤ 0.05. ^∗∗^Cluster – level *P*_*uncorrected*_ ≤ 0.05.

We reasoned that if this activation is a reflection of the participants’ choice to defect because of anger directed at their partner, then there should be a significant correlation between percent signal change and cooperation in the anger condition, but not in the sympathy or neutral conditions. Furthermore, the subjective rating from the self-report emotion questionnaire for anger words should correlate with the percent signal change. In fact, Pearson’s correlation coefficient showed a positive correlation between interaction contrast percentage signal change in the left putamen and the cooperation rate in the anger condition cooperation trials (*r* = 0.45, *p* = 0.045, respectively; Figure 3B). Furthermore, anger emotion strength as measured with the self-report emotion questionnaire negatively correlated with percent signal change in the left putamen (*r* = −0.50, *p* = 0.047). The correlation between anger emotion word ratings and the behavioral cooperation was negative but not significant (*r* = −0.30, *p* = 0.207).

Finally, there was no significant correlation between defection/cooperation and interaction contrast percent signal change in the left putamen in the neutral condition (*r* = −0.08, *p* = 0.751). Correlations between cooperation/defection in the anger condition and percent signal change in the left cingulate gyrus (BA 23), as well as between the cooperation/defection in the neutral condition and the percent signal change in the neutral condition in the left putamen, and the left posterior cingulate gyrus (BA 23) were not significant (*r* < 0.25, *p* > 0.288).

The reverse contrast [neutral (defection-cooperation) – anger (defection-cooperation)] did not show any significant activations.

#### Sympathy and Neutral Interaction With Defection and Cooperation Choice

**FIGURE 4:**
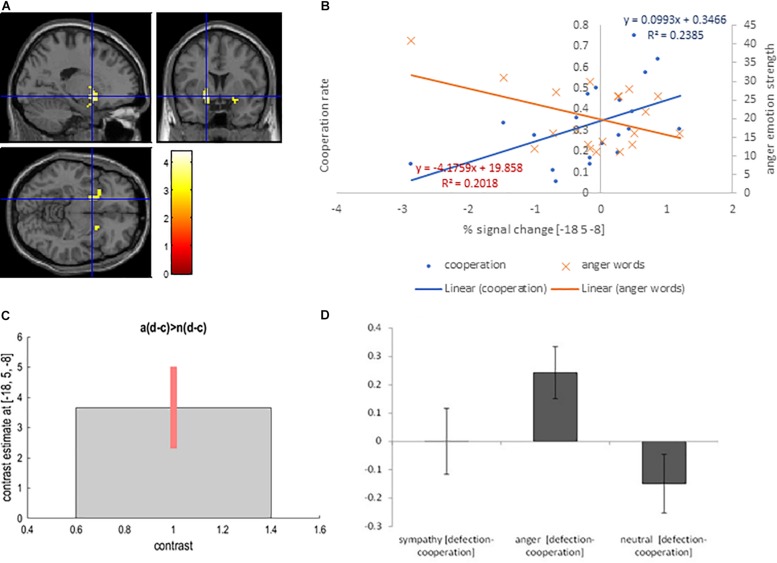
**(A)** Left amygdala activation overlaid on the MNI single subject template based on the interaction contrast [sympathy (defection – cooperation) – neutral (defection-cooperation)]; **(B)** Correlation between the interaction contrast percent signal change in the left amygdala and the cooperation rate in the sympathy condition (blue), and correlation between the percent signal change in the left putamen and the strength of sympathy word rating in the sympathy condition (orange); **(C)** Contrast estimate and 90% Confidence Interval for interaction contrast; **(D)** Difference in percent signal change between defection and cooperation trials in sympathy, anger and neutral emotion condition for interaction contrast.

The sympathy condition results in increased levels of cooperation. To isolate the neural basis of increased cooperation (decreased defection) in the sympathy condition we utilized the following contrast: [sympathy (defection – cooperation) – neutral (defection - cooperation)]. The contrast revealed activation in the right superior temporal pole (BA 28) (cluster level *P*_FWE_ < 0.05), and activation in the left amygdala (cluster level *P*_uncorrected_ < 0.05; Table 2 and Figures 4A,C,D).

Again correlation analyses were performed to test for a relationship between the cooperation in the sympathy and neutral emotion conditions in the activated areas. The left amygdala interaction contrast percent signal change positively correlated with cooperation in the sympathy condition (*r* = 0.57, *p* = 0.009; Figure 4B). In addition, the increase in the interaction contrast percent signal change in the left amygdala was positively correlated with self-report scores for sympathy words (*r* = 0.46, *p* = 0.043). In contrast, the positive correlation between self-report words and cooperation was not significant (*r* = 0.28, *p* = 0.234). Correlations between percent signal change in the left amygdala and the corresponding decisions were not significant (*r* ≤ 0.37, *p* ≥ 0.110) in the neutral emotion condition. The correlation in the right superior temporal pole and the left putamen with the defection in the sympathy and neutral conditions were not significant (*r* < 0.33, *p* > 0.15).

The reverse contrasts [neutral (defection – cooperation) – sympathy (defection – cooperation)] showed no significant activation.

#### Sympathy and Anger Interaction With Defection and Cooperation Choice

Finally, we examined the response by emotion (sympathy and the anger) interaction, [anger (defection – cooperation) – sympathy (defection – cooperation)], revealing activation in the left putamen, cluster level- *P*_uncorrected_ ≤ 0.05. The reversed contrast [sympathy (defection-cooperation) – anger (defection-cooperation)] showed activation in the left medial frontal gyrus (BA 10) (Cluster level- *P*_uncorrected_ ≤ 0.05).

The correlation between percent signal change in the left putamen with cooperation in the anger condition was a trend (*r* = 0.44, *p* = 0.052), while in the defection trials and in the sympathy condition cooperation and defection trials correlation was not significant (*r* ≤ −0.36, *p* ≥ 0.12). The left middle frontal gyrus (BA 10) activation in the anger and the sympathy emotion conditions did not correlate with the defection rate neither in defection nor in cooperation trials (*r* ≤ −0.38, *p* ≥ 0.10).

## Discussion

The current study investigated the functional neuroanatomy of cooperation and defection responses in the Prisoner’s Dilemma game under conditions of partner directed sympathy, anger, or neutral emotions. The outcome of the game was presented after each run (36 trials with three opponents) and did not provide information about what the opponents received. The reputation building was induced prior to the Prisoner’s Dilemma game via the emotion induction task. The behavioral results confirmed the effectiveness of the manipulation. As expected, participants’ cooperation rates increased significantly from the neutral to the sympathy condition and decreased from the neutral to the anger condition (trend level). Consistent with this, the sympathy condition elicited quicker responses for cooperation than defection choices, whereas this pattern was reversed in the anger and neutral conditions. Imaging results showed (relative) greater activation in the left putamen, in the anger condition, and in left amygdala, in the sympathy condition, compared to the neutral condition, in response to cooperation choices.

Left putamen percent signal change positively correlated with cooperation rate. Furthermore, self-reported anger emotion strength was negatively correlated with percent signal change in this area. These results suggest that relative increase in left putamen activation corresponds to more cooperative behavior, and given the negative correlation with self-report anger words strength, the putamen activation may be important for overcoming the desire to retaliate.

Previous studies have documented the role of striatum, and in particular, left putamen, in emotion regulation. In one study, participants were shown emotionally neutral faces and asked to engage either in positive emotion reappraisal (think positively about the face) or negative emotion reappraisal (think negatively) (Richey et al., 2015). Left putamen activation was observed during positive reappraisal trials. Furthermore, not only reappraisal, but also emotion suppression elicits activation in the left (and also right) putamen. Vanderhasselt et al. (2013) asked participants to view negative and high arousing images and either suppress the emotion or engage in negative emotion reappraisal. Negative emotion suppression, but not reappraisal, showed increased bilateral putamen activation. This is consistent with our suggestion that the putamen activation in the anger condition may be linked to overcoming the emotion and cooperating despite the anger directed at the partner.

The contrast sympathy (defection – cooperation) – neutral (defection – cooperation) showed activation in the left amygdala. This activation was positively correlated with both cooperation rates and self-reported sympathy emotion ratings: higher amygdala activation related to higher cooperation rates and higher scores on self-report sympathy emotion strength. These findings suggest that relative activation of the amygdala in the sympathy condition corresponds to increased cooperating responses and the use of more sympathy words to describe the participant. The finding is consistent with past studies. In the Singer et al. (2004) study, a cooperative opponent in the Prisoner’s Dilemma triggered sympathetic responses from the participants (as revealed by the post-trial questionnaire). Furthermore, intentional decision to cooperate by the opponent in this study was associated with increased amygdala activation; the left amygdala was activated when participants were presented with a photo of an intentional cooperator (person who decided to cooperate themselves instead of being assigned this decision by a computer). In a study investigating incidental fear during the Trust Game with social (with a human opponent) and non-social (decisions generated by computer) trials, results showed that a strong unexpected electrical shock (Threat of Shock, ToS) can reduce trust transfer rates in both social and non-social conditions (Engelmann et al., 2019). Furthermore, in the absence of ToS, a significant connectivity was observed between the temporo parietal junction (TPJ) and amygdala during social trust trials, but this connectivity was disrupted by the introduction of ToS. The authors suggest that the TPJ-amygdala connectivity present when there are no aversive emotional stimuli reflects information pre-processing occurring both cognitively (i.e., mentalizing, TPJ) and emotionally (trustworthiness of the opponent assessment, amygdala). However, once threatening stimuli are introduced, this connectivity is broken: the amygdala shows suppression and therefore breaks its communication with the TPJ, reducing one’s ability to mentalize. This suggests that increased amygdala activity indicates not only of emotional stimuli preprocessing, but also shows mentalizing processes. In another study, participants making altruistic decisions (cooperation) as opposed to selfish decisions (defection) also showed amygdala activation in the Prisoner’s Dilemma game (Cutler and Campbell-Meiklejohn, 2019). Increased amygdala activation might have been the result of participants not expecting their cooperation to be reciprocated: opponent’s unreciprocated cooperation toward participants’ resulted in increased activation of participants left amygdala (Rilling et al., 2008). Another explanation might come from the research exploring hippocampus and amygdala connectivity in episodic emotional memories (Phelps, 2004). Participants under the condition of receiving instructed anticipated emotional stimuli (indication of possible electric shock) showed increased left amygdala activation. This suggests that episodic memories can influence an individual’s emotional reactions in part by modulating amygdala activation. In the current study, it is possible that participants had episodic memories about the sympathy-triggering stimuli and experienced sadness toward the other. Therefore, they were possibly anticipating to feel guilty if they would choose defection and this resulted in the choice of cooperation and showed an increased left amygdala activation during these choices.

Additionally, the amygdala is part of the human reinforcement expectancies system which is involved in learning the signs of distress of others and in this way guiding individuals from antisocial behavior (Ray et al., 2005; Blair, 2007) and helping to solve moral dilemmas (Greene et al., 2004). As anticipatory emotions can guide individuals from antisocial behavior (Rick and Loewenstein, 2008), expectation of the guilt arising from their decision results in higher cooperation rates, which is in line with the withdrawal emotion function (Engelmann and Hare, 2018). Incidental sadness, which is related to sympathy emotion in our study, does not show the same reward processing as in the neutral emotion condition (Harlé et al., 2012). Furthermore, at the neural level, researchers found that the left ventral striatum showed stronger activation in the neutral condition (indicating reward processing) but in the sad condition, this pattern was not observed. Behaviorally, sad participants had a stronger preference toward fair offers during social interactions. Therefore, it is possible to assume that in our sympathy condition, participants were proposing fairer decisions in the Prisoner’s Dilemma game.

One unexpected finding was that the decrease in cooperation rates from neutral to anger was only a trend (Eimontaite et al., 2013). One possible explanation for the lack of significance may be that participants were medical personnel. Compassion and empathy are desirable skills in nurses and health care workers as they need to interpret and understand the feelings of their patients as well as demonstrate compassion for their condition (Morse, 1991) in addition to being able to restrain negative actions, remain calm and in control of their behavior in a stressful situation (Zhang et al., 2001). Due to these professional characteristics, the participants might have shown a strong response toward the partner in the sympathy condition, and may have been able to control their negative emotions in the anger condition. However, this requires further exploration.

Furthermore, not having actual reimbursement for the Prisoner’s Dilemma game might make the participant feel like the interactions are without real consequences and could be criticized on the grounds of not including choices with real outcomes and consequences. This could potentially have led to a stronger cooperation response in the sympathy condition compared to defection in anger. The results should be explored further within an environment where participants are reimbursed based on their decisions. Furthermore, the current study used an extent *k* = 20 and peak level *p* = 0.005 (uncorrected) combination threshold. This threshold was comparable to the Family Wise Error-corrected thresholds of Lieberman and Cunningham (2009), and Lieberman et al. (2009). Further discussion on the subject by Eklund et al. (2016) considered clusterwise thresholds to be invalid, and the results of the current study can be considered, therefore, exploratory. Finally, the small sample size is a limitation of the current study in terms of correlation results. Yarkoni (2009) argues that small sample size correlations results in power issues. A solution for this issue is a recommended increase in sample size (*N* > 50) (Yarkoni, 2009).

The current study investigated the effect of emotions on the iterated Prisoner’s Dilemma game, with the outcome of multiple interactions with three opponents revealed only after 36 trials, thus avoiding reputation building through game, and reputation building was only induced through emotion manipulation before participants performed on the Prisoner’s Dilemma game. The results show that the effects of partner directed sympathy and anger emotions on decision-making are represented by modulation of activation in the left putamen and the left amygdala. In particular, increased (relative) activation in left putamen is associated with increased cooperation decisions, even in the face of partner directed anger. Left amygdala activation increased (relatively) in response to increased number of cooperation responses in the partner sympathy directed condition. In addition, reaction times increased for decisions where participants went against their emotional impulse, providing further support, showing the conflict between emotional and rational. These results are important as they provide further evidence for the role of the left putamen and left amygdala in social exchange decision-making under the influence of partner directed emotion, yet without reputation building effects.

## Data Availability

The datasets generated for this study are available on request to the corresponding authors.

## Ethics Statement

This study was carried out in accordance with the recommendations of the University of Hull (United Kingdom) and the IRCCS San Camillo (Italy) with written informed consent from all subjects. All subjects gave written informed consent in accordance with the Declaration of Helsinki. The protocol was approved by the departmental ethics committee, University of Hull (United Kingdom) and the IRCCS San Camillo (Italy).

## Author Contributions

IE, MDM, and DD performed the measurements. VG, IS, and AV were involved in planning and supervised the work. IE processed the experimental data, performed the analysis, drafted the manuscript, and designed the figures. VG aided in interpreting the results and worked on the manuscript. All authors discussed the results and commented on the manuscript.

## Conflict of Interest Statement

The authors declare that the research was conducted in the absence of any commercial or financial relationships that could be construed as a potential conflict of interest.
