# Supplementary material for: Left Amygdala and Putamen Activation Modulate Emotion Driven Decisions in the Iterated Prisoner’s Dilemma Game
Source: Front Neurosci. 2019 Jul 17;13:741. doi: 10.3389/fnins.2019.00741 (PMC6650534; doi:10.3389/fnins.2019.00741)
Supplement: Supplementary file 1 [file Table_1.docx]

Supplementary Material

# **Self-Report Emotion Questionnaire**

This questionnaire adapted from Harmon-Jones, Peterson, and Vaughn (2003) and Harmon-Jones and Sigelman (2001) was used to evaluate whether the induction of the target emotion was successful. The questionnaire presented a list of 52 emotion adjectives assessing the following emotions: sympathy and compassion (compassionate, sympathetic, warm, moved, soft-hearted; n = 4), sadness for the other (sad, upset, feeling low, grieved, heavy-hearted, low spirited, sorrowful, guilty, troubled, worried, nervous, distressed, alert; n = 13), fear (afraid, jittery, scared , perturbed; n = 4), anger (agitated, irritable, angry, frustrated, hostile, furious, enraged, mad, alert, determined, bothered, alarmed, disgusted; n = 13) and neutral, slightly positive words (good mood, happy, active, enthusiastic, excited, pleasant, inspired, interested, proud, attentive, strong, glad, joyful, satisfied, pleased, calm, content, tranquil; n = 18).

Based on the definition of sympathy and anger emotions (Winterich, Han, & Lerner, 2010; Wispé, 1986), words being semantically related to sympathy, compassion, and sadness were pooled into a sympathy word group (Cronbach’s Alpha 0.914, n = 17). Similarly, words indicative of anger and fear emotions were combined to an anger emotion word list (Cronbach’s Alpha 0.875, n = 17). Finally, the neutral emotion word list contained adjectives associated with positive affect (Cronbach’s Alpha 0.834, n = 18). For each word, participants had to indicate how strongly they felt that particular emotion towards each of the three other “participants” on a scale from 1 (not at all) to 5 (very strong). The mean rating for each group of words was calculated for the analysis.

# **Emotion manipulation results**

The Self-Report Emotion Questionnaire was used to evaluate the success of emotion induction. A mixed 3 x 3 *ANOVA* with the within-subject factors Emotion Condition (sympathy, anger and neutral) and Word Group (sympathy, anger, neutral) was carried out. The effect sizes were calculated as suggested by Lakens (2013). The results showed significant main effects of Emotion Condition (*F*(2, 38) = 4.79, *p* = 0.014, *η_p_²* = 0.201), Word Group (*F*(2, 38) = 9.39, *p* < 0.001, *η_p_²* = 0.331) and a significant Emotion Condition by Word Group interaction (*F*(4, 76) = 9.15, *p* < 0.001, *η_p_²* = 0.325).

As the main interest of this analysis was to identify interactions between Emotion Condition and Word Group, the follow-up of the Emotion Condition by Word Group interaction with paired *t*-tests showed that, as expected, in the sympathy emotion condition the mean rating for the sympathy word group was higher compared to anger and at a trend level compared to neutrals words (*t*(19) = 5.64, *p* < 0.001, *d_z_* = 1.261, and *t*(19) = 2.37, *p =* 0.029, *d_z_* = 0.530, respectively; Bonferroni corrected alpha = 0.017, Table 1). Also, neutral words had significantly greater scores compared to anger words (*t*(19) = 2.85, *p =* 0.010, *d_z_* = 0.637). In the anger condition, the mean ratings for anger words trended higher compared to sympathy word ratings (*t*(19) = 2.36, *p* = 0.029, *d_z_* = 0.528), but there were no differences between anger and neutral or sympathy and neutral word groups (*t*(19) ≤ 2.06, *p* ≥ 0.054, *d_z_* ≤ 0.461). Finally, in the neutral emotion condition the neutral word group was rated significantly higher than anger words (*t*(19) = 4.59, *p* < 0.001, *d_z_* = 1.026) and at a trend difference compared to sympathy (*t*(19) = 2.51, *p* = 0.022, *d_z_* = 0.561). In addition, sympathy words showed a higher mean rating than anger words in this emotion condition (*t*(45) = 2.54, *p* = 0.02, *d_z_* = 0.568).

Supplementary Table 1: Emotion word groups mean scores (SD) as a function of the emotion condition

|  | Emotion condition | | |
| --- | --- | --- | --- |
|  | Sympathy | Neutral | Anger |
| Sympathy words group | 2.17 (0.75) | 1.43 (0.41) | 1.41 (0.40) |
| Neutral words group | 1.71 (0.42) | 1.88 (0.55) | 1.68 (0.58) |
| Anger words group | 1.37 (0.33) | 1.25 (0.34) | (0.49) |

### 3 Imaging results. The main effect of choice

To investigate what brain activation patterns are involved while participants choose to defect or to cooperate, the cooperation and the defection trials were contrasted with each other.

The main effect of choice revealed that in the contrast defection > cooperation the left caudate is activated. The reversed contrast shows the activation in the left inferior frontal gyrus (BA 46), the right middle frontal (BA 9) and the right precentral gyrus (BA4). This contrast also revealed a significant activation in the left insula (BA 13) and the right inferior frontal gyrus (BA 40; *P_FWE_* < .05 cluster level; Table 2).

Supplementary Table 2: The increased brain activation in the cooperation and defection comparison (at *p* = 0.01, *k* = 10, cluster level)

| Brain Region | | Brodmann Area | Hemisphere | # of voxels | peak T | MNI coordinates | | |
| --- | --- | --- | --- | --- | --- | --- | --- | --- |
|  |  |  |  |  |  | x {mm} | y {mm} | z {mm} |
| **cooperation>defection** | |  |  |  |  |  |  |  |
| Sub-lobar | |  |  |  |  |  |  |  |
|  | Insula | BA 13 | L | 56 | 5.10 | -42 | -4 | -5 |
| Frontal Lobe | |  |  |  |  |  |  |  |
|  | Inferior Frontal Gyrus | BA 46 | L | 14 | 3.39 | -45 | 39 | 16 |
|  | Middle Frontal Gyrus | BA 9 | R | 14 | 3.95 | 36 | 29 | 34 |
|  | Precentral Gyrus | BA 4 | R | 33 | 4.40 | 39 | -19 | 46 |
| Parietal Lobe | |  |  |  |  |  |  |  |
|  | Inferior Parietal Lobule | BA 40 | R | 76 | 3.35 | 53 | -31 | 34 |
| **defection>cooperation** | |  |  |  |  |  |  |  |
| Sub-lobar | |  |  |  |  |  |  |  |
|  | Caudate |  | L | 19 | -3.67 | -3 | 14 | 10 |

These decision-making areas are affected by the emotion, so the same contrast was performed in the neutral emotion trials only (Table 3). In this comparison defection did not show any significant activation but the cooperation showed a stronger activation in the right inferior frontal lobule (BA 40), the right superior temporal pole (BA 28), however, only in the left claustrum the activation was significant (*P_FWE_* < .05, cluster level; Table 3).

Supplementary Table 3: The increased brain activation in the cooperation and defection in the neutral emotion condition (at *p* = 0.01, *k* = 10, cluster level)

| Brain Region | | Brodmann Area | Hemisphare | # of voxels | peak T | MNI coordinates | | |
| --- | --- | --- | --- | --- | --- | --- | --- | --- |
|  |  |  |  |  |  | x {mm} | y {mm} | z {mm} |
| **cooperation> defection** | |  |  |  |  |  |  |  |
| Parietal Lobule | |  |  |  |  |  |  |  |
|  | Inferior Parietal Lobule | BA 40 | R | 16 | 4.19 | 51 | -34 | 40 |
| sub-lobar | |  |  |  |  |  |  |  |
|  | Claustrum |  | L | 37 | 4.23 | -30 | 2 | 13 |
| Limbic Lobe | |  |  |  |  |  |  |  |
|  | Superior Temporal Pole | BA 28 | R | 15 | 3.67 | 27 | 5 | -23 |
